# Botanical aspects, phytochemicals, and toxicity of *Tamarindus indica* leaf and a systematic review of antioxidant capacities of *T. indica* leaf extracts

**DOI:** 10.3389/fnut.2022.977015

**Published:** 2022-09-20

**Authors:** Sontaya Sookying, Acharaporn Duangjai, Surasak Saokaew, Pochamana Phisalprapa

**Affiliations:** ^1^UNIt of Excellence on Clinical Outcomes Research and Integration (UNICORN), School of Pharmaceutical Sciences, University of Phayao, Phayao, Thailand; ^2^Division of Pharmacy and Technology, Department of Pharmaceutical Care, School of Pharmaceutical Sciences, University of Phayao, Phayao, Thailand; ^3^Center of Health Outcomes Research and Therapeutic Safety (Cohorts), School of Pharmaceutical Sciences, University of Phayao, Phayao, Thailand; ^4^Department of Physiology, School of Medical Sciences, University of Phayao, Phayao, Thailand; ^5^Division of Pharmacy Practice, Department of Pharmaceutical Care, School of Pharmaceutical Sciences, University of Phayao, Phayao, Thailand; ^6^Division of Ambulatory Medicine, Department of Medicine, Faculty of Medicine Siriraj Hospital, Mahidol University, Bangkok, Thailand

**Keywords:** *Tamarindus indica*, antioxidant, *in vitro*, toxicity, phytochemicals, botanical aspects

## Abstract

Oxidative stress is a condition occurs when there is the imbalance between prooxidants and free radicals. It involves in cellular metabolism, aging, and immune response. Recently oxidative stress has been proved about its beneficial roles in human body. However, long term oxidative stress and high concentration of free radicals can lead to negative effects on organs, systems, and physiological conditions. Prooxidant or antioxidant, therefore, is one of the most important choices for the prevention of these anomaly. *Tamarindus indica* is a medicinal plant that has been reported as a source of antioxidants. The plants' leaves possess antioxidant effects according to many studies. However, these results have not yet been systematically summarized. The present systematic review summarizes and discusses about the *in vitro* antioxidant capacities of *T. indica* leaves. The plants' description and morphology, elements and phytochemical constituents, total phenolic and flavonoids contents and toxicity are also summarized and discussed here.

## Introduction

Oxidative stress refers to the imbalance between the production of free radicals in the body and the capability of cells and tissues to clear them (1). Free radicals are generated from endogenous and exogenous sources by enzymatic and non-enzymatic reactions. They play crucial roles in human health. Free radicals, such as nitric oxide radical (NO^•^) and superoxide radical (${\text{O}}_{2}^{•-}$), are involved in the defense mechanism to fight pathogens, the syntheses of some cellular structures, and cellular signaling pathways. In addition, they control blood flow by being cell-to-cell messengers, and they are required for non-specific host defense and induction of a mitogenic response (1). Thus, regular exposure to free radicals is one of the cellular homeostasis. Despite their benefits, free radicals can also contribute to the anomaly by being pro-oxidant. Long-term and high concentrations of free radicals are undesirable phenomenon (2). Oxidative stress occurs when there are excessive and rising levels of free radicals and oxidants in the body. Uncontrolled conditions lead to health problems and eventually increase the risk of metabolic, chronic, and degenerative diseases, such as cardiovascular diseases, neurodegenerative disorders, nephropathy, inflammation and immune-related diseases, sexual maturation and fertility disorders, and cancers (1, 3–7).

Oxidative stress is caused by excessive oxidants and a lack of antioxidants. Antioxidants refer to compounds able to impede or retard the oxidation of a substrate, acting at a lower concentration compared with that of the protected substrate (8). Antioxidants can be both endogenous and exogenous substances, similar to oxidants. Endogenous antioxidants are classified as enzymatic and non-enzymatic antioxidants. Exogenous antioxidants are introduced to the body in the form of a diet, and they act as oxidative defenses through different mechanisms and in different cellular compartments (6). Antioxidants such as vitamin C and E, coenzyme Q10, zinc and selenium, and polyphenols are sometimes inadequately consumed through routine diets. They have therefore sometimes been applied in the forms of dietary supplements or additive substances in foodstuffs.

Phytochemicals that are well-known as antioxidants are polyphenols, vitamins, carotenoids, minerals, and organosulfur compounds (9). There is plenty of research looking for the sources of powerful antioxidants due to their promising benefits for health from either their preventive or treatment perspectives. Thus, many plants that contain the aforementioned phytochemicals have been examined for their antioxidant activities and developed as sources of natural exogenous antioxidants.

*Tamarindus indica* L. (Fabaceae, Caesalpinioideae), or tamarind, is a tropical plant native to Africa. The plant has long been used as a food and herbal medicine. Its fruit pulp is well-known as a good source of vitamins, minerals, and organic acids. Tamarind fruit possesses several pharmacological activities, such as antifungal, antiasthmatic, hepatoprotective, and wound healing activities (10–13). Moreover, other parts of this plant, such as its leaf, stem bark, root bark, and seed, have also been reported as medicaments, e.g., antibacterial, antihyperlipidemic, antiulcer, anticancer, antifungal, wound healing, hepatoprotective and immunopotentiation agents (10, 11, 13–19).

There are various chemical constituents in *T. indica*. The fruit pulps contain furan derivatives, carboxylic acid, phlobatannin, grape acid, apple acid, flavonoids, pectin, sugars, and the like (20, 21). The seeds contain campesterol, β-amyrin, β-sitosterol, fatty acids, tannins, sugars, mucilage and polysaccharides, cardiac glycosides, and phenolics, among others (20, 22, 23). The components of the bark include tannins, saponins, glycosides, peroxidase, and lipids (20). The leaves contain orientin, iso-orientin, vitexin, iso-vitexin, glycosides, peroxidase, vitamin B_3_, and vitamin C (20, 23). Polyphenols, e.g., flavonoids and phenolics, are present in almost every part of the plant, making *T. indica* an up-and-coming source of antioxidative agents.

In this review article, we describe original research on the antioxidant activities of *T. indica*, focusing on antioxidant effect of its leaves obtained from the *in vitro* experiments (antioxidant capacity) (24). The description and morphology of *T. indica*, major chemical constituents especially phenolic compounds and flavonoids, and toxicity of *T. indica* leaves were also summarized and discussed. *T. indica* leaf extracts possessed antioxidant capacity by free radicals scavenging, heavy metal chelating and transition. Total phenolic and total flavonoids contents might relate to antioxidant capacity of *T. indica* leaves. The elements in *T. indica* leaves might also be responsible for the antioxidant capacity. No toxic was reported from the using of *T. indica* leaves either *in vitro* or *in vivo* experiments. The limitations of this study are lack of *in vivo* antioxidant activity assay, standard compounds were applied in only some included studies, no specific chemical was reported as biomarker and no quantification analysis of active compounds was conducted, maturity level of raw materials used in the included studies was reported in only one study.

## Methods

### Data sources and search strategy

Two authors (SSa and SS) independently searched electronic databases (EMBASE, PubMed, Scopus, Thai Journal Online Database, Thai Thesis Database, Science Direct, and Clinical Key). Relevant articles were searched from inception to April 2022. The strategic search terms were “*Tamarindus indica*” AND [(“leaves”) OR (“leaf”)] AND “antioxidant.” We also searched references in literature reviews and manuscripts published in journals. No limitations were placed on language or study design. In addition, we contacted the related researchers and experts for details and explanations of the articles.

### Study selection

**Figure 1:**
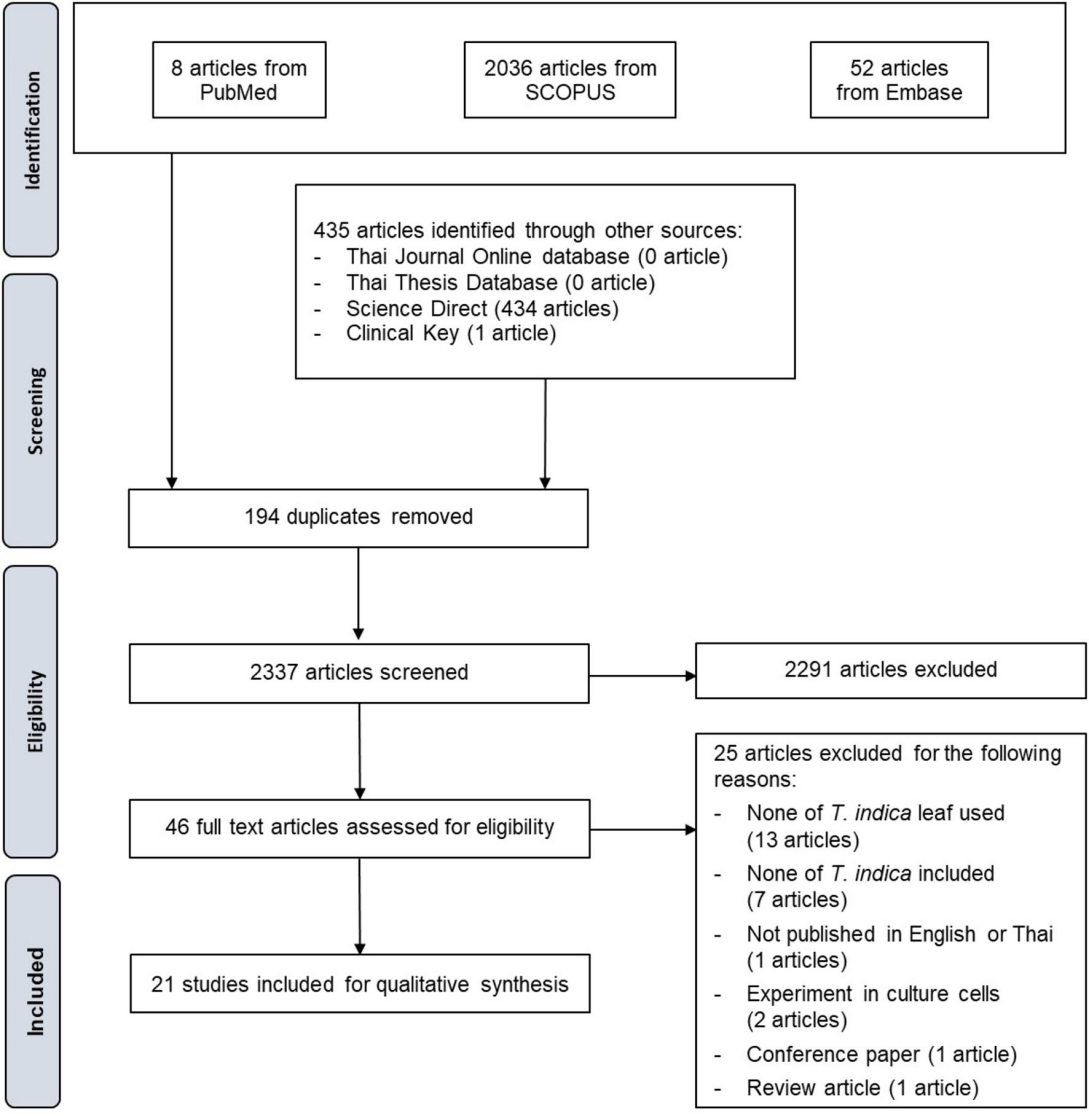
PRISMA flow diagram of the study selection process.

The studies included in this systematic review were selected according to the PRISMA guideline (Figure 1). After searching for articles, we removed duplicates, screened titles, and abstracts, and obtained the full texts of each article. We included research classified as (1) studies of the antioxidant capacity of *T. indica* leaves and (2) studies reporting measured outcomes (antioxidant capacity). A bibliographic search was then performed to identify articles from conference proceedings for which the full text was available. We excluded articles whose data had been obtained from prior studies. Accepted articles were included in this systematic review. Two investigators independently conducted the assessments. Twenty-one research articles from 7 databases were included. In all studies, 10 assays were used for the determination of antioxidant capacity. Five studies reported the results of phytochemical screening tests and elemental analyses. Seventeen studies revealed the quantity of total phenolics and total flavonoids, which are the major compounds responsible for *T. indica's* antioxidant capacity.

### Outcome measures

The primary outcome of interest was measures of the antioxidant capacity of *T. indica* leaves. The secondary outcome was the total phenolic or total flavonoid content of *T. indica* leaves, and the correlation between the total phenolic or total flavonoid content of *T. indica* leaves and their antioxidant capacities, if applicable.

### Data extraction

Two investigators independently reviewed each abstract and its associated full text. Each investigator also extracted data from each study for inclusion in the analysis. Data extraction was performed on study designs (part used, extract used, method and assay, and outcomes) and quality of studies Risk of bias was assessed using SciRAP with adaptation as a tool (25). The aspects of funding and competing interests were not focus in this study. In the report quality assessment, 1 item of test compound and controls, 2 item of test system, 3 items of administration of test compound, and 3 items of data collection and analysis were evaluated. In the methodological quality assessment, 3 items, 1 item, 1 item, and 3 items in the same aspects were evaluated, respectively. The results were reported as fulfilled, partially fulfilled, not fulfilled, and not determined. The latter was selected if the data was not available. Discrepancies were resolved by consensus.

### Data synthesis and analysis

The statistical heterogeneity was analyzed using *I*^2^ and *X*^2^ tests. Percentage *I*^2^ was identified based on the following equation: *I*^2^ = 100% (Q-*df*)/Q, where Q is Cochran's heterogeneity statistic and *df* is the degree of freedom. The heterogeneity was determined as “might not be important,” “may represent moderate heterogeneity,” “may represent substantial heterogeneity,” and “considerable heterogeneity” by the ranges of 0–40, 30–60, 50–90, and 75–100%, respectively (26). For the *X*^2^ test, a *P*-value of < 0.1 (significant) was used to assess heterogeneity.

## Results

### Study selection

In all, 2,960 identified studies were systematically searched, and 435 studies were identified through other sources (434 from Science Direct and 1 from Clinical Key). No articles were identified through the Thai Journal Online database or the Thai Thesis Database. After 194 duplicates were removed, 2,033 studies remained. Of these, 2,291 were discarded based on a review of their titles and abstracts. Forty-six articles were then assessed for eligibility. Twenty-five were discarded (13 for none of *T. indica* leaf used, 7 for none of *T. indica* included, 2 for experiment conducted in cultured cells, 1 for not published in English or Thai, 1 conference paper, and 1 review article), leaving 21 for inclusion in the qualitative analysis (Figure 1). In the quantitative analysis, the included data had high levels of heterogeneity. The I^2^ values of each data set classified as antioxidant capacity assay and outcome measure were all higher than 75% (92.0–99.4%). Therefore, a meta-analysis was not conducted.

### Study characteristics

**Figure 2:**
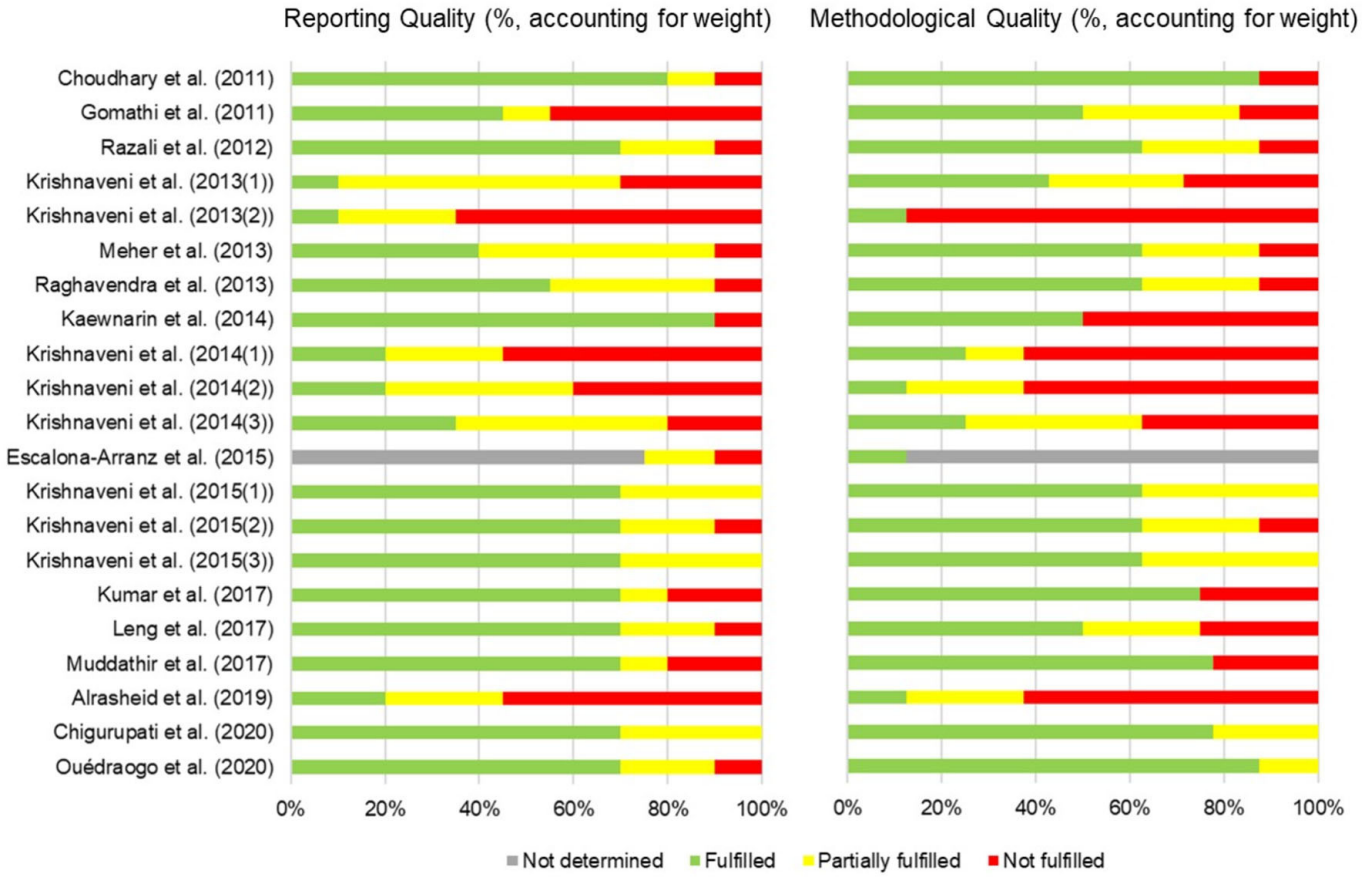
Risk of bias summary assessment of included studies. The bars represent the reporting quality and methodological quality of individual studies resulting from the average of the quality of (1) test compound and controls (2) test system (3) administration of test compound and (4) data collection and analysis. The evaluation used SciRAP with adaptation as a tool (25).

The characteristics of all 21 studies are summarized in Table 1. Risk of bias were shown in Figure 2. The results obtained from the evaluation of 4 aspects e.g., test compound and controls, test system, administration of test compound, and data collection and analysis. The plant materials were *T. indica* leaves with different pretreatments and untreated leaves before the extraction was manipulated. Fresh leaves were used in 9 studies, while the other studies used dried leaves that had been oven dried, air dried, shade dried or stir fried. The extraction solvents used were acetone, ethyl acetate, hexane, methanol, ethanol, and water. The extraction methods varied between studies. They were maceration, Soxhlet extraction, hot extraction, fluid extraction, cold percolation, and fresh preparation using a mortar and pestle. Five studies did not report their extraction method. Ten assays were used to determine the antioxidant capacity of *T. indica* leaf extracts. Even though the outcome measures differed between studies, the 1,1-diphenyl-2-picrylhydrazyl (DPPH^•^) radical scavenging and ferric ion reducing antioxidant power (FRAP) assays were the most commonly employed methods. Other assays used were metal chelating (ferrous [Fe^2+^] ion chelating [FIC]), nitric oxide (NO^•^) radical scavenging, total antioxidant capacity (phosphomolybdenum), 2,2'-azino-bis (3-ethylbenzthiazoline-6-sulfonic acid) (ABTS^•+^) radical scavenging, hydrogen peroxide (H_2_O_2_) scavenging, superoxide radical (${\text{O}}_{2}^{•-}$) scavenging, hydroxyl radical (HO^•^) scavenging, and β-carotene bleaching (BCB) assays. No sample concentration or dose was provided in 2 studies (34, 45). The standard positive controls used in the assays were the universal antioxidants, i.e., ascorbic acid, quercetin, rutin, butylated hydroxyanisole (BHA), and butylated hydroxytoluene (BHT). However, a positive control was not determined in some studies. Phytochemical screening tests were conducted in 4 studies (Table 2). The total polyphenol and/or total flavonoid contents of *T. indica* leaf extracts were established in 17 studies, and 4 investigations reported correlations between antioxidant contents and antioxidant capacities (Table 3).

**Table 1:** Study characteristics of the *in vitro* antioxidant capacity of *T. indica* leaf extracts.

**References**	**Plant material**	**Solvent**	**Extraction method**	**Antioxidant capacity assay**	**Dose/concentration**	**Outcome (unit)**	**Results (mean ± SD)**	**Remarks**
Choudhary and Swarnkar (27)	Air-dried leaves (Temp: T_room_)	Methanol	Maceration	DPPH^•^ radical scavenging	1,000 μg/ml	Scavenging capacity (%)	16.80*	Positive control: BHT = 68.20
				${\text{O}}_{2}^{•-}$ radical scavenging	1,000 μg/ml	Anion scavenging capacity (%)	31.86 ± 3.11	Positive control: BHT = 81.19 ± 3.43
Gomathi et al. (28)	Air-dried leaves (Shade dried) (Temp: N/A)	Acetone	Soxhlet extraction	DPPH^•^ radical scavenging	N/A	IC_50_ (μg/ml)	171.00 ± 2.40	Positive control: BHT = 37.80 ± 0.80 BHA = 29.00 ± 1.2 0
		Methanol	Soxhlet extraction				124.70 ± 2.10	
		Water	Maceration				283.10 ± 1.10	
		Acetone	Soxhlet extraction	HO^•^ radical scavenging	N/A	IC_50_ (μg/ml)	66.60 ± 2.10	Positive control: BHT = 7.80 ± 2.70 BHA = 12.30 ± 4.30
		Methanol	Soxhlet extraction				46.90 ± 2.20	
		Water	Maceration				79.20 ± 1.50	
		Acetone	Soxhlet extraction	FIC	N/A	Ferrous ion chelating capacity (mg EDTA Equivalent/g extract)	71.50 ± 0.60	Positive control: BHT = 143.07 ± 1.80 BHA = 192.10 ± 2.30
		Methanol	Soxhlet extraction				79.70 ± 1.20	
		Water	Maceration				64.30 ± 2.40	
		Acetone	Soxhlet extraction	BCB	250 μg	Peroxidation inhibitory capacity (%)	48.30 ± 0.70	Positive control: BHT = 67.8 ± 0.7 BHA = 80.9 ± 1.8
		Methanol	Soxhlet extraction				17.50 ± 0.10	
		Water	Maceration				11.30 ± 2.10	
Razali et al. (29)	Air-dried leaves (Temp: N/A)	Methanol	Maceration	DPPH^•^ radical scavenging		Antioxidant capacity (mmol TE/g dried weight)	3.17 ± 0.00	Positive control: Rutin = 3.32 ± 0.00 Quercetin = 3.60 ± 0.00
		Ethyl acetate	Maceration		25–100 μg/ml		2.76 ± 0.03	
		Hexane	Maceration				1.35 ± 0.04	
		Methanol	Maceration	FRAP	N/A	Ferric reducing capacity (mmol/g dried weight)	1.87 ± 0.09	Positive control: Rutin = 3.36 ± 0.003 Quercetin = 13.30 ± 0.002
		Ethyl acetate	Maceration				0.57 ± 0.9	
		Hexane	Maceration				0.12 ± 0.07	
		Methanol	Maceration	ABTS^•+^ radical scavenging	100–2,000 μg/ml	Antioxidant capacity (mmol TE/g dried weight)	1.65 ± 0.04	Positive control: Rutin = 1.72 ± 0.01 Quercetin = 4.18 ± 0.03
		Ethyl acetate	Maceration				0.70 ± 0.01	
		Hexane	Maceration				0.51 ± 0.03	
		Methanol	Maceration	${\text{O}}_{2}^{•-}$ radical scavenging	25–400 μg/ml	Anion scavenging capacity (mmol TE/g dried weight)	4.64 ± 0.003	Positive control: Rutin = 5.47 ± 0.01 Quercetin = 5.67 ± 0.004
		Ethyl acetate	Maceration				4.54 ± 0.14	
		Hexane	Maceration				3.99 ± 0.01	
Krishnaveni et al. (30)	Fresh leaves	Water	N/A	FRAP	Equivalent to 10 mg fresh leaves	Antioxidant capacity (mg AAE/g extract)	2.25*	
				FIC	Equivalent to 10 mg fresh leaves	Ferrous ion chelating capacity (mg EDTA Equivalent/g extract)	3.50*	
				NO^•^ radical scavenging	Equivalent to 10 mg fresh leaves	Antioxidant capacity (mg QE/g extract)	1.22*	
				Total antioxidant capacity	Equivalent to 10 mg fresh leaves	Total antioxidant capacity (mg AAE/g extract)	29.40*	
Krishnaveni et al. (31)	Fresh leaves	Water	N/A	FRAP	N/A	Antioxidant capacity (mg AAE/g extract)	2.45*	
				FIC	N/A	Ferrous ion chelating capacity (mg EDTA Equivalent/g extract)	4.70*	
				NO^•^ radical scavenging	N/A	Antioxidant capacity (mg QE/g extract)	1.10*	
				Total antioxidant capacity	N/A	Total antioxidant capacity (mg AAE/g extract)	27.30*	
Meher and Dash (32)	Air-dried leaves (Shade dried) (Temp: N/A)	Water	Hot extraction	DPPH^•^ radical scavenging	50–500 μg/ml	IC50 (μg/ml)	346.63*	Positive control: Ascorbic acid = 56.70
		Ethanol	Maceration				301.83*	
		Water	Hot extraction	HO^•^ radical scavenging	50–500 μg/ml	IC_50_ (μg/ml)	346.63*	Positive control: Ascorbic acid = 56.70
		Ethanol	Maceration				292.04*	
		Water	Hot extraction	FRAP	500 μg/ml	Reducing power (FRAP value)	0.33 ± 0.03	Positive control: Ascorbic acid = 2.00
		Ethanol	Maceration				0.76 ± 0.08	
		Water	Hot extraction	NO^•^ radical scavenging	50-500 μg/ml	IC_50_ (μg/ml)	339.35*	Positive control: Ascorbic acid = 77.31
		Ethanol	Maceration				279.90*	
Raghavendra et al. (33)	Air-dried leaves (Shade dried for 1 week) (Temp: N/A)	Methanol	Soxhlet extraction	DPPH^•^ radical scavenging	N/A	IC_50_ (μg/ml)	210.00*	Positive control: Ascorbic acid = 6.80
				ABTS^•+^ radical scavenging	N/A	IC_50_ (μg/ml)	35.00*	Positive control: Ascorbic acid = 13.70
				Total antioxidant capacity	100-500 μg/ml	Total antioxidant capacity (μg/ml AAE)	72.00*	
Kaewnarin et al. (34)	Oven-dried leaves (Temp: 50°C) (Young leaves)	Ethyl acetate	Maceration	DPPH^•^ radical scavenging	N/A	Inhibitory capacity (%)	23.40 ± 1.80	Positive control: N/A
		Ethanol	Maceration				17.60 ± 1.10	
Krishnaveni et al. (35)	Fresh leaves	Water	N/A	FRAP	N/A	Antioxidant capacity (mg AAE/g extract)	≈7.50–9.00	
				FIC	N/A	Antioxidant capacity (mg AAE/g extract)	≈4.50–5.50	
				NO^•^ radical scavenging	N/A	Antioxidant capacity (mg QE/g extract)	≈7.00–11.50	
				Total antioxidant capacity	N/A	Total antioxidant capacity (mg AAE/g extract)	≈3.00–7.00	
Krishnaveni et al. (36)	Fresh leaves	Water	N/A	FRAP	N/A	Antioxidant capacity (mg AAE/g extract)	7.30*, 2.32*, 8.60*	Raw materials were obtained from 3 different sources
				FIC	N/A	Antioxidant capacity (mg AAE/g extract)	5.12*, 2.70*, 7.22*	Raw materials were obtained from 3 different sources
				NO^•^ radical scavenging	N/A	Antioxidant capacity (mg QE/g extract)	8.68*, 6.90*, 13.80*	Raw materials were obtained from 3 different sources
				Total antioxidant capacity	N/A	Total antioxidant capacity (mg AAE/g extract)	5.60*, 6.76*, 6.08*	Raw materials were obtained from 3 different sources
Krishnaveni et al. (37)	Fresh leaves	Water	N/A	FRAP	N/A	Antioxidant capacity (mg AAE/g extract)	3.10 ± 0.05	
				FIC	N/A	Antioxidant capacity (mg AAE/g extract)	2.60 ± 0.27	
				NO^•^ radical scavenging	N/A	Antioxidant capacity (mg QE/g extract)	4.60 ± 0.38	
				Total antioxidant capacity	N/A	Total antioxidant capacity (mg AAE/g extract)	2.50 ± 0.10	
Escalona-Arranz et al. (38)	Air-dried leaves	Water	Fluid extraction	DPPH^•^ radical scavenging	N/A	IC_50_ (μg/ml)	44.36 ± 3.72	Positive control: Quercetin = 10.88 ± 0.81
				FRAP	N/A	IC_50_ (μg/ml)	60.87 ± 1.07	Positive control: Quercetin = 21.94 ± 0.80
				FIC	N/A	Estimated binding constant (mol/l)	1.09*	Positive control: Quercetin = 2.000
Krishnaveni et al. (39)	Fresh leaves	Water	Fresh preparation using mortar and pestle	FRAP	Equivalent to 10 mg fresh leaves	Antioxidant capacity (mg AAE/g extract)	2.81 ± 0.49	
				FIC	Equivalent to 10 mg fresh leaves	Antioxidant capacity (mg AAE/g extract)	3.33 ± 0.63	
				NO^•^ radical scavenging	Equivalent to 10 mg fresh leaves	Antioxidant capacity (mg QE/g extract)	4.83 ± 2.45	
				Total antioxidant capacity	Equivalent to 10 mg fresh leaves	Total antioxidant capacity (mg AAE/g extract)	3.40 ± 1.12	
				H_2_O_2_ scavenging	Equivalent to 10 mg fresh leaves	H_2_O_2_ scavenging capacity (%)	2.13 ± 0.45	
Krishnaveni et al. (40)	Fresh leaves	Water	Fresh preparation using mortar and pestle	FRAP	Equivalent to 10 mg fresh leaves	Antioxidant capacity (mg AAE/g extract)	2.95 ± 0.08	
				FIC	Equivalent to 10 mg fresh leaves	Antioxidant capacity (mg AAE/g extract)	2.90 ± 0.34	
				NO^•^ radical scavenging	Equivalent to 10 mg fresh leaves	Antioxidant capacity (mg QE/g extract)	3.41 ± 0.57	
				Total antioxidant capacity	Equivalent to 10 mg fresh leaves	Total antioxidant capacity (mg AAE/g extract)	0.98 ± 0.20	
				H_2_O_2_ scavenging	Equivalent to 10 mg fresh leaves	H_2_O_2_ scavenging capacity (%)	3.00 ± 0.48	
Krishnaveni et al. (41)	Fresh leaves	Water	Fresh preparation using mortar and pestle	FRAP	Equivalent to 10 mg fresh leaves	Antioxidant capacity (mg AAE/g extract)	3.00 ± 0.86	
				FIC	Equivalent to 10 mg fresh leaves	Antioxidant capacity (mg AAE/g extract)	4.03 ± 0.98	
				NO^•^ radical scavenging	Equivalent to 10 mg fresh leaves	Antioxidant capacity (mg QE/g extract)	3.55 ± 0.25	
				Total antioxidant capacity	Equivalent to 10 mg fresh leaves	Total antioxidant capacity (mg AAE/g extract)	2.10 ± 0.08	
				H_2_O_2_ scavenging	Equivalent to 10 mg fresh leaves	H_2_O_2_ scavenging capacity (%)	4.05 ± 0.66	
Kumar et al. (42)	Air-dried leaves (Shade dried)	Methanol	Cold percolation	DPPH^•^ radical scavenging	50 μg/ml	Scavenging capacity (%)	28.58 ± 1.14	Positive control: Ascorbic acid = 96.50 ± 0.19
					100 μg/ml		39.43 ± 0.77	Positive control: Ascorbic acid = 96.45 ± 0.11
					200 μg/ml		61.70 ± 1.90	Positive control: Ascorbic acid = 96.67 ± 0.17
					300 μg/ml		77.36 ± 1.07	Positive control: Ascorbic acid = 96.25 ± 0.17
					400 μg/ml		87.56 ± 1.17	Positive control: Ascorbic acid = 96.25 ± 0.17
					500 μg/ml		91.39 ± 1.22	Positive control: Ascorbic acid = 96.49 ± 0.16
Leng et al. (43)	Fresh leaves	Methanol	Maceration	DPPH^•^ radical scavenging	Equivalent to 2 mg fresh leaves	Inhibitory capacity (%)	16.458 ± 1.53	Positive control: N/A
	Oven-dried leaves (At 60°C for 3 h)	Methanol	Maceration		Equivalent to 2 mg oven-dried leaves		39.028 ± 0.25	Dose: Positive control: N/A
	Stir fried leaves (stir fried using kitchen stove at 180°C for 10 min)	Methanol	Maceration		Equivalent to 2 mg stir fried leaves		69.923 ± 0.11	Positive control: N/A
Muddathir et al. (44)	Air-dried leaves (Shade dried) (Temp: T_room_)	Methanol	Maceration	FRAP	1,000 μg/ml	Ferric reducing ability of plasma (mM FE/mg dried weight)	2.71 ± 0.06	Positive control: Quercetin = 3.96 ± 0.11 Ascorbic acid = 3.79 ± 0.10 BHT = 2.84 ± 0.03
Alrasheid et al. (45)	Air-dried leaves (Temp: N/A)	Ethanol	Maceration	DPPH^•^ radical scavenging	N/A	Scavenging capacity (%)	61.66*	Positive control: Ascorbic acid = 93.5
Chigurupati et al. (46)	Air-dried leaves (Mature and healthy leaves) (Shade dried)	Ethanol	Maceration	DPPH^•^ radical scavenging	1,000 μg /ml	IC_50_ (μg/ml)	1.42 ± 0.3	Positive control: Ascorbic acid = 1.09 ± 0.02
				ABTS^•+^ radical scavenging	1,000 μg/ml	IC_50_ (μg/ml)	1.62 ± 0.66	Positive control: Ascorbic acid = 1.02 ± 0.03
Ouédraogo et al. (47)	Air-dried leaves (Shade dried) (Temp: T_room_)	Water	Maceration	DPPH^•^ radical scavenging	3,750 μg/ml	Antioxidant capacity (μmol AAE/g extract)	360.02 ± 7.23	Positive control: Quercetin = 646.00 ± 0.00
				FRAP	100 μg/ml	Antioxidant capacity (μmol AAE/g extract)	677.26 ± 24.53	Positive control: Quercetin = 6034.64 ± 12.05
				ABTS^•+^ radical scavenging	100 μg/ml	Antioxidant capacity (μmol AAE/g extract)	7067.58 ± 0.00	Positive control: Quercetin = 14550.26 ± 281.08

AAE, ascorbic acid equivalent; ABTS, 2,2'-azino-bis (3-ethylbenzthiazoline-6-sulfonic acid); BCB, β-carotene bleaching; BHA, butylated hydroxyanisole; BHT, butylated hydroxytoluene; DPPH, 1,1-diphenyl-2-picrylhydrazyl; EDTA, ethylene diamine tetraacetic acid; FE, ferrous equivalent; FIC, ferrous ion chelating; FRAP, ferric reducing antioxidant power; H_2_O_2_, hydrogen peroxide; N/A, data not available; NO, nitric oxide; QE, quercetin equivalent; SD, standard deviation; TE, Trolox equivalent; Temp, temperature; T_room_, room temperature; ^*^ no SD available.

**Table 2:** Phytochemical screening of crude *T. indica* leaf extracts.

**Phytochemicals**	**Raghavendra et al. (33)**	**Kumar et al. (42)**	**Alrasheid et al. (45)**	**Chigurupati et al**. **(****46****)**	
	**Methanolic extract**	**Methanolic extract**	**Ethanolic extract**	**Ethanolic extract**	**Methanolic extract**
Flavonoids	–	N/A	–	N/A	N/A
Alkaloids	+	+	+/–	+	+
Tannins	N/A	N/A	+/–	+	+
Saponins	N/A	–	+	+	+
Steroids	–	N/A	+	+	+
Terpenoids	N/A	N/A	–	N/A	N/A
Coumarin	N/A	N/A	–	N/A	N/A
Glycosides	+	N/A	+/–	+	+
Phenolics	–	+	N/A	N/A	N/A
Monosaccharides	N/A	N/A	N/A	+	+
Carbohydrates	N/A	+	+	+	+
Reducing sugars	N/A	N/A	–	–	–
Non-reducing sugars	N/A	N/A	N/A	–	–
Amino acids	N/A	–	N/A	–	–
Proteins	N/A	–	N/A	+	+
Mucilage and gums	N/A	N/A	N/A	+	+
Lignins	N/A	N/A	+	N/A	N/A

+, positive; –, negative; +/–, negative and positive presented in different tests; N/A, data not available.

**Table 3:** Total polyphenol and total flavonoid contents of *T. indica* leaf extracts.

**References**	**Total phenolic content**	**Total flavonoid content**	**Remarks**
Choudhary and Swarnkar (27)	4.72 ± 0.08 mg GAE/g DW methanolic extract	1.06 ± 0.08 mg QE/g DW of methanolic extract	N/A
Gomathi et al. (28)	33.10 ± 4.00 mg GAE/g acetone extract 26.80 ± 2.10 mg GAE/g methanolic extract 16.01 ± 1.60 mg GAE/g aqueous extract	74.10 ± 1.10 mg QE/g acetone extract 24.30 ± 2.30 mg QE/g methanolic extract 7.30 ± 5.20 mg QE/g aqueous extract	Regression correlation coefficient: Total phenolic content with antioxidant capacity (r^2^) in assays • DPPH^•^ scavenging = 0.211 • HO^•^ scavenging = 0.580 • Metal chelating = 0.720 • BCB = 0.482
Razali et al. (29)	309.00 ± 3.78 mg GAE/g methanolic extract 101.00 ± 12.26 mg GAE/g ethyl acetate extract 31.8 ± 3.70 mg GAE/g hexane extract	N/A	Regression correlation coefficient: Total phenolic content with antioxidant capacity (r) in assays • FRAP = 0.8899 • DPPH^•^ scavenging = 0.8849 • ABTS^•+^ scavenging = 0.8264
Krishnaveni et al. (30)	1.10 mg GAE/g water extract	9.70 mg QE/g water extract	N/A
Krishnaveni et al. (31)	0.10 mg GAE/g water extract	3.00 mg QE/g water extract	N/A
Raghavendra et al. (33)	20.00 mg GAE/g methanolic extract	410.00 mg QE/g methanolic extract	N/A
Kaewnarin et al. (34)	0.29 ± 0.00 mg GAE/g ethyl acetate extract	130.00 ± 3.90 mg QE/g ethyl acetate extract	Pearson correlation coefficient (r): •Total phenolic content with DPPH^•^ scavenging capacity = 0.866 • Total flavonoid content with DPPH^•^ scavenging capacity = 0.583
	0.15 ± 0.00 mg GAE/g ethanolic extract	69.30 ± 1.70 mg QE/g ethanolic extract	Pearson correlation coefficient: • Total phenolic content with DPPH^•^ scavenging capacity = 0.779 • Total flavonoid content with DPPH^•^ scavenging capacity = 0.796
Krishnaveni et al. (35)	≈5.00–5.50 mg GAE/g water extract	≈3.50–5.50 mg QE/g water	N/A
Krishnaveni et al. (36)	6.70 mg GAE/g water extract	8.00 mg QE/g water extract	N/A
Krishnaveni et al. (37)	6.10 ± 0.40 mg GAE/g water extract	6.60 ± 0.30 mg QE/g water extract	N/A
Krishnaveni et al. (39)	3.53 ± 2.02 mg GAE/g water extract	5.93 ± 2.36 mg QE/g water extract	N/A
Krishnaveni et al. (40)	7.23 ± 2.36 mg GAE/g water extract	2.20 ± 0.00 mg QE/g water extract	N/A
Krishnaveni et al. (41)	4.63 ± 2.19 mg GAE/g water extract	4.16 ± 0.05 mg QE/g water extract	N/A
Leng et al. (43)	39.31 ± 1.34 mg GAE/g methanolic extract of fresh leaves	N/A	Regression correlation coefficient: Total phenolic content with antioxidant capacity (r^2^) = 0.877
	47.74 ± 1.78 mg GAE/g methanolic extract of oven-dried leaves		
	139.87 ± 2.22 mg GAE/g methanolic extract of stir fried leaves		
Muddathir et al. (44)	31.26 ± 0.38 mg GAE/g methanolic extract	N/A	N/A
Chigurupati et al. (46)	1.80 mg GAE/g ethanolic extract (maceration)	1.44 mg RUE/g ethanolic extract (maceration)	N/A
	1.01 mg GAE/g ethanolic extract (Soxhlet extraction)	1.04 mg RUE/g ethanolic extract (Soxhlet extraction)	
Ouédraogo et al. (47)	202.40 ± 1.50 mg GAE/g water extract	99.00 ± 1.20 mg QE/g water extract	N/A

DW, dried weight; GAE, gallic acid equivalent; N/A, data not available; QE, quercetin equivalent; RUE, rutin equivalent.

### Antioxidant capacity of *T. indica* leaves

A summary of the antioxidant capacities of *T. indica* leaf extracts is given in Table 1. Approximately 10 assays were used to determine antioxidant capacity. In each assay, some studies determined the antioxidant capacity using the same measurement, while some other investigations used different methods. The results obtained from each method are summarized in the following section.

#### DPPH (DPPH^•^) radical scavenging

The DPPH^•^ radical scavenging assay is a free radical scavenging antioxidant assay. The principle of the method is the reaction between antioxidant and an organic radical. The method has high sensitivity. The results are comparable to those of other free radical scavenging assays and are reproducible. The assay can be applied for the quantitative analysis of complex biological samples. Another advantage of DPPH^•^ radical scavenging assays is correlation with bioactive compounds (phenols, flavonoids) with regression factor (R) > 0.8. Although the DPPH^•^ radical scavenging assay can be performed easily, the DPPH^•^ radical is a synthetic radical that cannot represent the i*n vivo* system (48). More than this, the levels of antioxidants needed for scavenging these radicals are not physiologically possible nor relevant.

To determine the DPPH^•^ radical scavenging capacity of *T. indica* leaves, acetone, methanol, water, and ethanol were used to extract the air-dried leaves of *T. indica*. In the study of Gomathi et al. (28), it was found that acetone extract had better antioxidant capacity than methanol and water extracts (IC_50_ values of 171.00, 124.70, and 283.10 μg/ml, respectively). These values correlate with the finding of Meher and Dash (32) that ethanolic extract was more potent than water extract (IC_50_ values of 301.83 and 346.63 μg/ml, respectively). Aqueous extracts were used in the studies by Gomathi et al. (28), Meher and Dash (32), and Escalona-Arranz et al. (49). They reported that fluid extraction gave the highest antioxidant effect compared with maceration and hot extraction techniques. Ethanolic extracts obtained from maceration by Meher and Dash (32) and Chigurupati et al. (46) expressed IC_50_ values of 5.3- and 1.3-fold that of ascorbic acid, respectively, as a positive control. The methanolic extract obtained from cold percolation extraction by Kumar et al. (42) exhibited scavenging capacities of 28.6–91.4% in a concentration-dependent manner (50–500 μg/ml). The macerated-aqueous extract produced by Ouédraogo et al. (47) gave antioxidant capacity equivalent to ascorbic acid 360.0 mg/g extract.

A study by Leng et al. (43) compared the difference between raw material pretreatment methods before extraction using methanol by the maceration technique. The results showed that the extract obtained from the stir-fried, oven-dried and fresh leaves offered 69.9, 39.0, and 16.5% inhibitory capacity, respectively (dose equal to 2 g fresh leaves). Pretreatment by oven-drying in Kaewnarin et al.'s (34) study showed that extraction using ethyl acetate offered higher inhibitory capacity than ethanol (23.4 and 17.0%, respectively).

Razali et al. (29) compared the antioxidant capacity of the extract obtained from air-dried leaves and maceration extraction using methanol, ethyl acetate, and hexane. It was found that the methanolic extract presented the highest capacity, followed by the ethyl acetate and hexane extracts [3.2, 2.8, 1.4 mmol Trolox equivalent (TE)/g dried weight, respectively].

#### ABTS (ABTS^•+^) radical scavenging

The ABTS radical scavenging assay is a free radical scavenging antioxidant assay based on the same principle as the DPPH^•^ scavenging assay. The assay also provides reproducible results and regression factor (R) > 0.8 with bioactive compounds (phenols, flavonoids). However, the limitation of the assay is that ABTS^•+^ radicals do not exist naturally; thus, the result cannot represent the *in vivo* system as well as a DPPH^•^ radical scavenging assay (48). More than this, the levels of antioxidants needed for scavenging these radicals are not physiologically possible nor relevant.

The ABTS^•+^ radical scavenging assay was performed in 4 studies. The IC_50_ values of the methanolic and ethanolic extracts were 35.0 μg/ml (ascorbic acid, 13.7 μg/ml) and 1.6 μg/ml (ascorbic acid, 1.0 μg/ml), respectively (33, 46). The results of the study by Razali et al. (29) showed that the methanolic extract obtained from maceration expressed antioxidant capacity close to that of the standard compound rutin (1.65 vs. 1.72 mmol TE/g dried weight), as ethyl acetate and hexane extracts possessed lower capacities (0.7 and 0.5 mmol TE/g dried weight, respectively). The ABTS^•+^ radical scavenging capacity determined in Ouédraogo et al.'s (47) study using aqueous extract was 7067.6 μmol AAE/g extract, which can be calculated as half of the positive control, quercetin (14550.2 μmol AAE/g).

#### Superoxide (${\text{O}}_{2}^{•-}$) radical scavenging

The superoxide radical scavenging assay is the assessment of antioxidants' ability to prevent ${\text{O}}_{2}^{•-}$ radical generation. The generation of ${\text{O}}_{2}^{•-}$ radicals generally occurs in the normal respiratory process. The ${\text{O}}_{2}^{•-}$ radical is then converted into H_2_O_2_, which is further converted into O_2_ and water. The assay resembles free radical production and quenching in the human body, and it is superior to the DPPH^•^ and ABTS^•+^ radical scavenging assays (48).

The ${\text{O}}_{2}^{•-}$ radical scavenging capacity of methanol, ethyl acetate, and hexane extracts of *T. indica* dried leaves was determined by Razali et al. (29). The results revealed that the radical scavenging capacity of the methanolic extract was better than that of the ethyl acetate and hexane extracts (4.6, 4.5, 4.0 mmol TE/g dried weight, respectively). These results correlated with those of DPPH^•^ radical scavenging capacity in the same study. The methanolic extract used in Choudhary and Swarnkar's (27) study showed a scavenging capacity of 31.9% at 1000 μg/ml.

#### Hydroxyl (HO^•^) radical scavenging

The hydroxyl radical is the most harmful reactive oxygen species (ROS) in the human body. It can lead to cell damage, cell apoptosis, and cell mutation by reacting with polyunsaturated fatty acid moieties. Hydroxyl (HO^•^) radical scavenging assays have been developed to determine lipid peroxidation in cells and tissues by HO^•^ radicals. The method was also used to measure the radical capacity of HO^•^ and antioxidants with slight modification. This method offers accurate results in most cases (48).

The HO^•^ radical scavenging capacity of *T. indica* leaf extracts was investigated in 2 studies. It was found that the air-dried leaf aqueous extracts obtained from maceration and hot extraction exhibited IC_50_ values of HO^•^ radical scavenging capacity of 79.2 and 346.6 μg/ml, respectively (Gomathi et al. (28), Meher and Dash (32)). The methanolic and acetone extracts showed better capacity, with IC_50_ values of 46.9 and 66.6 μg/ml, respectively, in the study of Gomathi et al. (28), similar to the ethanolic extract in the study of Meher and Dash (32) (IC_50_ = 292.0 μg/ml).

#### Ferric ion reducing antioxidant power

Ferric ion reducing antioxidant power is a reducing potential antioxidant assay. It is referred to as the ferric reducing ability of plasma. The FRAP assay is a method in which antioxidants react with a ferrous (Fe^3+^) complex, ferric-tripyridyltriazine [Fe^III^(TPTZ)]^3+^, forming an intense blue-colored ferrous complex [Fe^II^(TPTZ)]^2+^ under acidic conditions (pH 3.6). The strengths of the assay are its high sensitivity and reproducibility, its applicability to a broad spectrum of samples, and the correlation (R) with the H_2_O_2_ scavenging assay is > 0.8 (50). The limitation of the method is its non-specificity (48).

The FRAP assay was determined in 13 studies. One of these studies, Meher and Dash (32), reported the capacity as the μM ferric ion reduced to ferrous form per ml (FRAP value). The values were 0.3 and 0.8 for 500 μg/ml ethanolic and aqueous extracts, respectively, compared with 2.0 for ascorbic acid (positive control). Escalona-Arranz et al. (49) revealed an IC_50_ of 60.9 μg/ml water extract, while quercetin, the positive control, was 21.9 μg/ml. The ferric reducing ability of plasma was determined in the study of Muddathir et al. (44) using a methanolic extract, and the antioxidant capacity was 2.7 mM (ferrous equivalent FE)/mg dried weight. Another study described the reducing power in terms of ferric reducing capacity. The results were 0.1, 0.6, and 1.9 mmol/g dried weight for hexane, ethyl acetate, and the methanolic extract, respectively (29). The other studies focused on the capacity on the ascorbic acid equivalent. Eight studies conducted by Krishnaveni et al. resulted in 2.3–8.3 mg AAE/g extract (30, 31, 35–37, 39–41). Ouédraogo et al. (47) reported a value of 667.26 μmol AAE/g extract, which was ~10% of standard quercetin (6034.6 μmol AAE/g).

#### Ferrous ion chelation

The principle of the method is based on the oxidative stress caused by ROS originating from transition or heavy metals. Even if the method gives good reproducibility and repeatability, there are still limitations. They are (1) non-specific reactions (the assay not only reacts with phenolic compounds but also reacts with peptides and sulfate in the test medium); (2) the result obtained from the assay sometimes does not correlate with the total bioactive assays; and (3) poor correlation with FRAP, DPPH^•^, and ABTS^•+^ radical scavenging assays (48).

The results from FIC assays are summarized herein. Krishnaveni et al. (30) and Krishnaveni et al. (31) reported the ferrous ion chelating capacity of aqueous extracts as 3.5 and 4.7 mg ethylene diamine tetraacetic acid (EDTA) equivalent/g extract, respectively, which is very different from the study of Gomathi et al. (28) (64.3 mg EDTA equivalent/g extract). The acetone and methanolic extracts tested by Gomathi et al. (28) gave approximate results to the aqueous extract. The ferrous ion-chelating capacity of the aqueous extract determined by Escalona-Arranz et al. (49) was lower than that of the positive control quercetin (estimated binding constant = 1.1 vs. 2.0 mol/l). The antioxidant capacities of aqueous extracts obtained from fresh leaves determined in 6 studies by Krishnaveni et al. as the equivalent to ascorbic acid were in the range of 2.5–5.5 mg ascorbic acid equivalent (AAE)/g extract (35–37, 39–41).

#### β-carotene bleaching

The β-carotene bleaching assay determines the bleaching capability of antioxidants on β-carotene. The oxidized linoleic acid in an emulsion system is set to generate free radicals, leading to oxidative destruction of β-carotene. The rate of oxidative destruction is measured. The method can be applied to both lipophilic and hydrophilic samples. Nevertheless, it has some limitations similar to FIC assays (48).

There was only one study that investigated the β-carotene/linoleic acid peroxidation inhibitory capacity of *T. indica* leaf extracts (28). In this study, 250 μg of acetone, methanolic, and water extracts were applied. The peroxidation inhibitory capacities of the extracts were in the range of 11.3%−48.3%.

#### Nitric oxide (NO^•^) radical scavenging

NO^•^ radical is found in vascular endothelial cells. The radical is generated from an amino acid, L-arginine. The NO^•^ radical plays a vital role in the human body, and an excessive quantity of NO^•^ radicals can lead to several health complications. A nitric oxide radical scavenging assay was developed to determine the capability of antioxidants to scavenge NO^•^ radicals (48).

There were 9 studies that observed the NO^•^ radical scavenging capacity of *T. indica* leaf extracts. The IC_50_ values of ethanolic and aqueous extracts were determined by Meher and Dash (32). They were 3.6- and 4.4-fold that of the standard ascorbic acid, respectively (279.9 and 339.3 μg/ml vs. 77.3 μg/ml) (32). The scavenging capacities in the remaining 8 studies were determined by Krishnaveni et al. The values were in the range of 1.1–7.0 mg quercetin equivalent (QE)/g extract (30, 31, 35–37, 39–41).

#### Total antioxidant capacity

The total antioxidant capacity, or phosphomolybdenum assay, is the determination of the antioxidant capacity of the antioxidant sample to reduce molybdenum (VI) to molybdenum (V) or the formation of a phosphomolybdenum complex. The method can be applied to a wide spectrum of samples, but there are several limitations as well. They are (1) non-specific, (2) poorly correlated with bioactive compounds, and (3) poorly correlated with the results obtained from the DPPH^•^ radical scavenging assay (48).

The assays were performed in 8 studies by Krishnaveni et al. and one study by Raghavendra et al. Methanolic extract of *T. indica* leaf showed a total antioxidant capacity of 72.0 μg/ml calculated as ascorbic acid equivalent (33), while aqueous extracts showed capacities of 0.98–29.4 mg AAE/g extract (30, 31, 35–37, 39–41).

#### Hydrogen peroxide (H_2_O_2_) scavenging

Hydrogen peroxide is a major oxygen metabolite generated *in vivo* by activated phagocytes and oxidase enzymes. The H_2_O_2_ scavenging capacity of antioxidants is assessed based on a peroxidase system (48).

The H_2_O_2_ scavenging capacity of *T. indica* leaf extracts was determined in 3 experiments. The aqueous extracts obtained from 3 works of Krishnaveni et al. possessed H_2_O_2_ scavenging capacities of 2.1–4.1% at a dose of 10 mg fresh leaves (39–41).

### *T. indica* description and morphology

**Figure 3:**
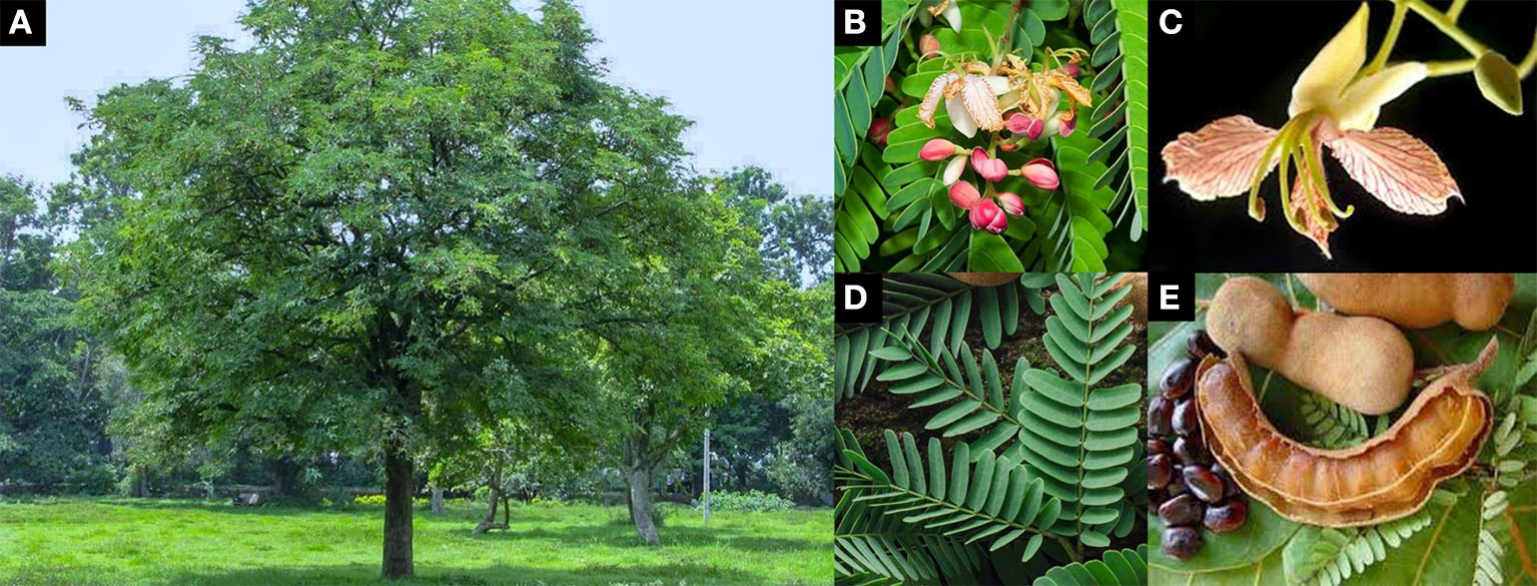
Characteristics of *T. indica* **(A)** habit **(B)** inflorescence **(C)** floret **(D)** leaves **(E)** ripe fruits and seeds [adapted from (51–55)].

*T. indica* belongs to the Fabaceae family and the Caesalpinioideae subfamily. The plant is an indigenous tropical evergreen tree up to 30 m in height, with a spreading crown up to 12 m in diameter. Leaves are unipinnate compound, 15 cm long, with an alternate arrangement. Young leaves are light green and become darker while maturing. Each leaf is composed of 10 to 18 pairs of opposite leaflets along the central axis, which close at night. Leaflets are narrowly oblong and sized 12–32 × 3–11 mm. The flowers are borne on inflorescences up to ~20 cm in length. The floret is 2.5 cm wide and has a caesalpiniaceous pattern, 4 sepals, and 5 petals (3 pale yellow petals with pinkish to red veins and 2 tiny thread-like petals). The fruits are pod- or legume-like, indehiscent, 10–18 × 4 cm, and straight or curved. The raw fruits are brown, and the fleshy inside is green-soft. Ripe fruits are brown with a soft and sticky pulp. There are 3 to 10 seeds, which are ~1.6 cm long, irregularly shaped, testa hard, shiny, and smooth (Figure 3) (51, 56).

### Phytochemistry of *T. indica*

Four out of the included studies provided the phytochemical screening results of the crude extract obtained from *T. indica* leaves (33, 42, 45, 46) (Table 2). Alkaloids were detected in ethanolic and methanolic extracts in all studies. Tannins, saponins, steroids, glycosides, monosaccharides, carbohydrates, mucilage, and gums were detected in both the methanolic and ethanolic extracts by Chigurupati et al. (46), while reducing and non-reducing sugars and amino acids were not present in either extract. Saponins and steroids were not detected in the methanolic extract of *T. indica* leaves by Raghavendra et al. (33) and Kumar et al. (42), which contrasts with those tested by Alrasheid et al. (45) and Chigurupati et al. (46). Proteins were not detected by Kumar et al. (42) but were detected by Chigurupati et al. (46). The absence of amino acids in the methanolic leaf extract tested by Kumar et al. (42) was confirmed by Chigurupati et al. (46). Although flavonoids and phenolics were not detected in the methanolic extract in the studies of Raghavendra et al. (33) and Alrasheid et al. (45), these compounds were quantitatively reported in the other studies presented in Table 3. Total phenolic and total flavonoid contents were quantitatively determined in 17 studies (Table 3). The phenolic contents were quantitatively determined as gallic acid equivalents, while the flavonoid contents were determined as quercetin or rutin equivalents. The total phenolic contents tended to be higher in the methanolic extract than in the samples extracted using ethanol, ethyl acetate, and hexane. However, the methanolic extract contained fewer phenolics and flavonoids than the acetone extract. The study of Leng et al. (43) indicated that the pretreatment method for plant materials significantly affects the total phenolic and flavonoid contents. The methanolic extract of stir-fried leaves had a significantly higher phenolic content than the methanolic extract of oven-dried leaves and fresh leaves. The difference in extraction methods, Soxhlet and maceration, in Chigurupati et al.'s (46) study showed non-significant results in total phenolic content. The results of Kaewnarin et al. (34) showed that either phenolics or flavonoids in ethyl acetate extract were 100% higher than those in ethanolic extract even though both samples were obtained with the maceration technique. The correlation between antioxidant capacities and total phenolic contents was analyzed in 4 studies, e.g., Gomathi et al. (28), Razali et al. (29), Kaewnarin et al. (34), and Leng et al. (43). Positive correlations were reported in all of them. Total flavonoid contents also had positive correlations with antioxidant capacities in the study by Kaewnarin et al. (34).

**Figure 4:**
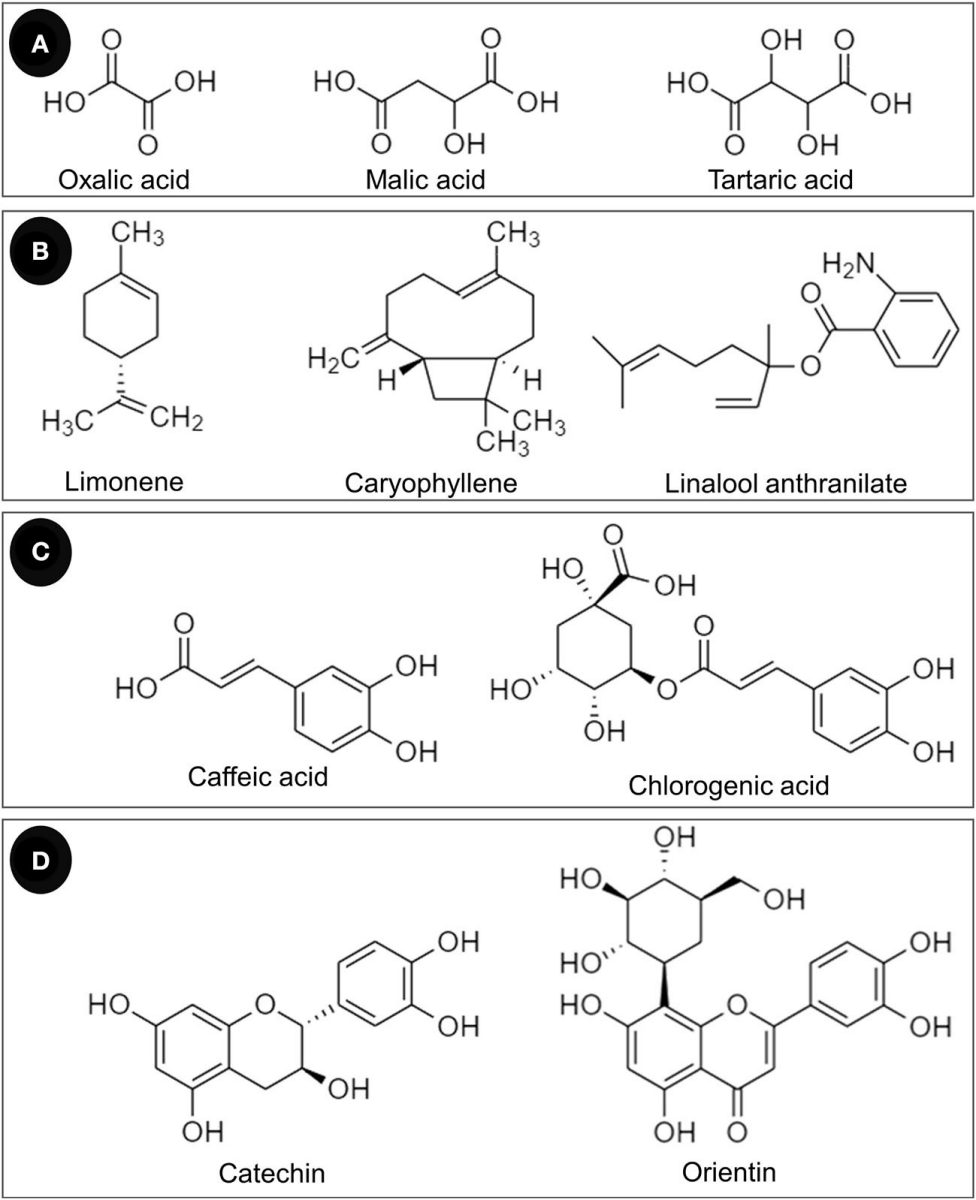
Chemical constituents of *T. indica* leaves classified as **(A)** organic acids, **(B)** terpenoids, **(C)** phenolic acids, and **(D)** flavonoids.

In addition to phenolic compounds and flavonoids, the chemical compositions of *T. indica* leaves and their relative abundance are detailed in Table 4. Some structures of chemical constituents of *T. indica* leaves are shown in Figure 4. The elements in *T. indica* leaf and leaf extracts investigated by Escalona-Arranz et al. (38) are presented in Table 5. Several classes of phytochemicals have been reported as constituents in *T. indica* leaves. Fatty acids, organic acids, terpenoids, tannins, flavonoids, and other organic compounds were reported to be found in *T. indica* leaf extracts. The type of solvent used in the extraction procedure provided different compositions and quantities.

**Table 4:** Major phytochemical compositions in *T. indica* leaves.

**Major constituents**	**% Relative abundance**	**References**
Oleic Acid	85.96 (ethanolic extract)	(57)
	39.00 (acetone extract)	
3-*O*-Methyl-d-glucose	43.09^*^ (ethanolic extract)	(58)
4-C-methyl-myo-inositol		
2-C-methyl-myo-inositol		
9-Octadecenoic acid (*E*)-, methyl ester (Methyl oleate)	41.05 (acetone extract)	(57)
*cis*-Vaccenic acid	35.23^*^ (aqueous extract)	(58)
*trans*-13-Octadecenoic acid		
Oleic Acid		
Benzyl benzoate	40.60 (leaf oil)	(59)
Limonene	24.40 (leaf oil)	(59)
	9.05 (chloroform extract)	(38)
3-Eicosyne	21.99 (n-hexane fraction obtained from ethanolic extract)	(38)
Tartaric acid	21.96 (chloroform fraction obtained from ethanolic extract)	(38)
	7.30 g/kg fresh weight (aqueous extract)	(60)
Octadecanoic acid	20.28^*^ (aqueous extract)	(58)
Octadecanoic acid, 2-(2-hydroxyethoxy) ethyl ester		
Eicosanoic acid		
Hexadecanoic acid (Palmitic acid)	20.99 (n-hexane fraction obtained from ethanolic extract)	(38)
	18.39 (chloroform fraction obtained from ethanolic extract)	(38)
	8.14 (ethanolic extract)	(57)
7,10-octadecadienoic, methyl ester	16.13 (n-hexane fraction obtained from ethanolic extract)	(38)
Malic acid	15.95 (chloroform fraction obtained from ethanolic extract)	(38, 60)
	0.75 g/kg fresh weight (aqueous extract)	(60)
9,12,15-octadecatrienoic acid, methyl ester	13.57 (n-hexane fraction obtained from ethanolic extract)	(38)
10-Octadecenoic acid	12.74 (n-hexane fraction obtained from ethanolic extract)	(38)
	7.77 (chloroform fraction obtained from ethanolic extract)	(38)
Hexadecanol (Cetyl alcohol)	12.4 (leaf oil)	(59)
6,10,14-trimethylpentadeca-5,9,13-trien-2-one	9.70 (n-hexane fraction obtained from ethanolic extract)	(38)
Benzene-1,2-dicarboxylic acid (Phthalic acid)	9.45 (chloroform fraction obtained from ethanolic extract)	(38)
2,2-dimethoxy-propane	8.93^*^ (ethanolic extract)	(58)
1,3-Dioxolane		
2-(1-methylethoxy)-ethanol		
Methyl-15-tricosanoate	8.39 (chloroform extract)	(38)
Pentadecanol	8.20 (leaf oil)	(59)
4-hydroxy-4-methyl-2-pentanone (Diacetone alcohol)	7.87^*^ (ethanolic extract)	(58)
2-methyl-2-hexanol		
N-methyl-ethanamine		
*n*-Nonadecanoic acid	7.57 (chloroform fraction obtained from ethanolic extract)	(38)
Longifolene	7.51 (chloroform extract)	(38)
*n*-Hexadecanoic acid (Palmitic acid)	7.40^*^ (aqueous extract)	(58)
L-Ascorbyl 2,6-dipalmitate		
Pentadecanoic acid		
Eicosane	7.34^*^ (aqueous extract)	(58)
1-Iodo-2-methylundecane		
10-Methylnonadecane		
2,6-di-tert-butyl-4-methylphenol (Butylated hydroxytoluene)	7.24 (chloroform extract)	(38)
Methyl palmitate	6.41 (chloroform extract)	(38)
	7.09 (acetone extract)	(57)
Caryophyllene	5.56 (chloroform extract)	(38)
Diphenyl-ether	5.47 (chloroform extract)	(38)
Cryptopinone	5.28 (chloroform extract)	(38)
Linalool anthranilate	4.70 (leaf oil)	(59)
	3.96 (chloroform extract)	(38)
Oxalic acid	7.50 g/kg fresh weight (aqueous extract)	(60)
Citric acid	1.00 g/kg fresh weight (aqueous extract)	(60)
Caffeic acid	N/A (butanol fraction obtained from ethanolic extract)	(38)
Luteolin	N/A (ethyl acetate fraction obtained from ethanolic extract)	(38)
Luteolin-7-*O*-glucoside	N/A (ethyl acetate fraction obtained from ethanolic extract)	(38)
Apigenin	N/A (ethyl acetate fraction obtained from ethanolic extract)	(38)
Orientin	N/A (butanol fraction obtained from ethanolic extract)	(38)
	N/A (methanolic and chloroform extract)	(61)
Iso-orientin (Homo-orientin)	N/A (butanol fraction obtained from ethanolic extract)	(38)
	N/A (methanolic and chloroform extract)	(61)
Vitexin	N/A (butanol fraction obtained from ethanolic extract)	(38)
	N/A (methanolic and chloroform extract)	(61)
Isovitexin (Saponaretin)	N/A (methanolic and chloroform extract)	(61)
Quercetin	N/A (ethyl acetate extract)	(29)
Isorhamnetin	N/A (hexane extract)	(29)
Catechin	N/A (methanol extract)	(29)
Epicatechin	N/A (methanolic, ethyl acetate, hexane extract)	(29)
3-*O*-Caffeoylquinic acid (Chlorogenic acid)	N/A (methanolic and chloroform extract)	(61)
4-*O*-Caffeoylquinic acid (Chlorogenic acid)	N/A (methanolic and chloroform extract)	(61)

* More than 1 composition in the same peak determined by GC–MS.

**Table 5:** Elements in *T. indica* leaf and leaf extracts (38).

**Elements**	***T. indica* leaf (μg/g)**	**Chloroform extract (μg/g)**	**Ethanolic extract (μg/g)**
Al	5.27	0.013	1.181
Cd	0.0019	–	–
Co	0.880	–	0.108
Cr	0.250	–	0.079
Cu	7.900	0.196	0.857
Fe	16.160	0.241	1.107
Mn	2.500	0.027	0.750
Ni	0.461	–	0.052
Pb	0.700	–	0.050
Sr	0.325	–	0.051
Zn	7.990	0.031	0.292
Mo	0.260	–	–
V	–	–	–
Se	4.723	0.083	1.341

### Toxicity of *T. indica* leaves

An acute oral toxicity study of ethanolic leaf extract of *T. indica* was conducted by Livingston Raja et al. (62) in albino Wistar rats. After receiving the extract at 1,000, 2,000, and 4,000 mg/kg body weight (BW) orally for 14 consecutive days, a non-significant difference in blood chemical parameters and adverse effects was observed (62). In 2015, the acute oral toxicity and oral mucous irritability of *T. indica* leaf fluid extract were determined in rats by Escalona-Arranz et al. (49). In an acute oral toxicity test of 2000 mg/kg BW, the extract was reported to be a non-toxic substance within the scale of toxic class substances (OECD/OCDE 423 2012) (49). The extract did not cause significant changes in hair and skin, mucous and eye color, histopathology of visceral organs, behavior, or somatomotor capacity. The assays were completed with a survival rate of 100%. The extract also did not change the macroscopic characteristics of Syberian hamsters after exposure to the right malar bag. However, it did show degeneration of the epithelium and mild vascular congestion in muscular tissue. The results correlated with the results obtained by Amado et al. (63). Amado et al. (63) tested the acute oral toxicity of dry *T. indica* leaf extract in male Wistar rats using the limit dose of 5000 mg/kg BW. It was found that at day 14 after administration, no death was observed. There were no significant differences between the treatment and control groups. There were no changes in skin and pelage, mucous membrane and eyes, or color and morphology of visceral organs (63). Moreover, the acute oral toxicity of ethanolic extracts of *T. indica* leaves was also investigated in healthy Sprague Dawley rats by Chigurupati et al. (46). No lethality or abnormal behavior was observed over the 14-day period after the administration of 2000 mg/kg BW (46).

The intraperitoneal acute toxicity (50% lethal dose) of 566 mg/kg BW aqueous leaf extract was reported in the study of Akor et al. (64). The extract showed moderate toxicity in Wistar albino rats (64).

The toxicity in erythrocytes of ethanolic and aqueous extracts of *T. indica* leaves was conducted by Mehdi et al. (65). It was found that neither extract induced hemolysis, similar to normal saline solution (65). These results agreed with a previous study performed by Escalona-Arranz et al. (66). The study of *T. indica* leaf fluid extract on human blood cells was conducted, and the results revealed that the extract did not cause significant hemolysis at 20–100 mg/ml. The protein denaturation ratio after the application of plant extracts at 40–100 mg/ml was very low compared with the control; thus, it was proven to be less toxic. In addition, the extract showed a protective effect against H_2_O_2_-induced oxidative damage in the human erythrocyte membrane at the same concentrations (66).

## Discussion

This systematic review examined 21 *in vitro* studies of the antioxidant capacity of *T. indica* leaves. The samples used in antioxidant tests were prepared using water or organic solvents by different methods and with different pretreatments of the raw materials. The antioxidant capacity assays also differed between studies.

Oxidative stress is the disruption of redox signaling and control caused by the imbalance of free radicals and antioxidant defenses (8). Free radicals are found in human cells, animal cells, and other living organisms. They are generated by endogenous reactions and are caused by exogenous sources. In the human body, free radicals are produced by several biochemical processes. For example, H_2_O_2_ and HO^•^ radicals result from the reduction of molecular oxygen during aerobic respiration, and ${\text{O}}_{2}^{•-}$ radicals and hypochlorous acid (HOCl) arise from the activation of phagocytes (67). Exogenous oxidants are caused by pollution and environmental stressors such as cigarette smoking, air pollution, radiation, and diet (8). Oxidative stress leads to a variety of health problems. It is both the primary cause of pathology and a secondary contributor to disease progression, e.g., cancers, cardiovascular disease, neurodegenerative disorders, diabetes, and metabolic syndrome (9). Thus, antioxidants are the first option to prevent and treat various health issues and anomalies, especially environmental pollution.

Antioxidants prevent oxidative stress-related damage by breaking radical chain reactions (9). Antioxidants are divided into endogenous and exogenous antioxidants. The latter class must be ingested through the diet. Endogenous antioxidants comprise enzymatic and non-enzymatic antioxidants, while exogenous antioxidants consist of water-soluble and lipid-soluble antioxidants. The well-known antioxidants obtained from natural sources are vitamin C and E, carotenoids, tannins, phenolic acids, and flavonoids (1, 8, 9).

Phenolics are strong antioxidants and are members of the “polyphenols,” which are a large class of plant secondary metabolites. Flavonoids are natural compounds that contain hydroxyl groups that are responsible for metal chelation and free radical scavenging capacity. The compounds can react with ${\text{O}}_{2}^{•-}$ and HO^•^ and are also able to chelate metal ions, so they regulate both iron homeostasis and redox state (9, 68). Pizzino et al. (1) summarized the antioxidant properties of flavonoids as ROS scavengers and ROS synthesis suppressors, antioxidant defense enhancers, enzyme inhibitors, and trace element chelators (1). Several flavonoids are present in *T. indica* leaf extracts, e.g., luteolin and its derivatives, apigenin, orientin, vitexin, quercetin, isorhamnetin, catechin, and epicatechin (Table 4). Non-flavonoid compounds such as caffeic acid and chlorogenic acid were also detected (Table 4).

Vitexin and iso-vitexin, which are apigenin derivatives, and orientin (the luteolin glycoside) were investigated for their *in vitro* antioxidant capacities and *in vivo* antioxidant activities. Khole et al. (69) studied the mechanism of vitexin and iso-vitexin for their antioxidant effects. They found that the compounds exhibited different capacities against ROS. Iso-vitexin scavenges ${\text{O}}_{2}^{•-}$ radicals better than vitexin, while vitexin scavenges NO^•^ radicals better. Both compounds were active against short-lived radicals: ABTS^•+^ radical and ${\text{CO}}_{3}^{•-}$ radical. These compounds protected HepG2 cells from H_2_O_2−_induced oxidative insult by modulating antioxidant enzyme levels and reducing intracellular ROS levels (69). In 2012, An et al. (70) observed the antioxidant activities of vitexin and orientin compared with vitamin E in an age mouse model. The results showed that vitexin and orientin had the capacity to improve the antioxidative system as well as to improve the levels of ATPase in the tissue and serum in aged mice induced by D-galactose. Furthermore, the compounds at 40 mg/kg BW were comparable to vitamin E for the improvement of neuronal cell structure and function in mice (70).

Catechin, epicatechin, rutin, and quercetin are ubiquitous polyphenols in herbs and food plants. These compounds showed better DPPH^•^ radical scavenging capacity than the analog of vitamin E, Trolox, in the study of Iacopini et al. (71). Similarly, in the DPPH^•^ scavenging, ABTS^•+^ radical scavenging, and FRAP assays performed by Tian et al. (72), quercetin manifested better antioxidant capacities than vitamin C and BHT in all assays. In a mechanistic study, quercetin exhibited several mechanisms against oxidative stress. It inhibited inducible nitric oxide synthase in macrophages, so oxidative damage was inhibited. The compound also directly scavenges free radicals and inhibits the formation of oxygen free radicals through the chelation of ions of transition metals such as iron. Quercetin also inhibited xanthine oxidase and suppressed TNF-α modulated by oxidative stress, resulting in the decrement of oxidative injury and the modulation of immune response (73).

Luteolin and apigenin are plant flavonoids with a broad spectrum of biological activities. Both displayed superior ABTS^•+^ radical scavenging capacity to vitamin C and BHT in the studies of Tian et al. (72). In addition, luteolin showed surpassing results in DPPH^•^ radical scavenging and FRAP assays compared with both vitamin C and BHT (72). The *in vitro* mechanism of apigenin against oxidative stress includes oxidative enzyme inhibition, modulation of redox signaling pathways (NF-κB, Nrf2, MAPK, and P13/Akt), reinforcement of enzymatic and non-enzymatic antioxidants, free radical scavenging, and metal chelation (74). An *in vivo* experiment in arterial aging mice conducted by Clayton et al. (75) revealed that apigenin could increase NO bioavailability; normalize ROS, antioxidant expression, and oxidative stress; and abolish the inhibitory effect of ROS (75). The mechanisms of the antioxidant action of luteolin have been summarized as ROS scavenging, ROS-generating oxidase inhibition, enhancement and protection of endogenous antioxidants, direct inhibition of oxidative-catalyzed enzymes, and chelation of transition metal ions (76).

Chlorogenic acid and its major metabolite, caffeic acid, are classified as phenolic acids. Chlorogenic acid is hydrolyzed into caffeic acid in the intestine after ingestion. The antioxidative effect of caffeic acid has been evinced using different *in vitro* assays by Gülçin (77), i.e., the ferric thiocyanate method, total reduction capability, ABTS^•+^ radical scavenging, DPPH^•^ radical scavenging, ${\text{O}}_{2}^{•-}$ radical scavenging, and ferrous metal chelating capacity (77). The *in vivo* antioxidant assay of chlorogenic acid and caffeic acid was performed using the 2-methyl-6-p-methoxyphenylethynylimidazopyrazynone method to emit ${\text{O}}_{2}^{•-}$ radical scavenging capacity. The IC_50_ values of chlorogenic acid and caffeic acid were 41.0 and 10.1 μM, respectively, whereas allopurinol provided an IC_50_ of 15.0 μM (78). Caffeic acid exerted its cytoprotective effect in ischemia/reperfusion injury in the rat small intestine caused by ROS. The compound decreased lipid peroxidation and reduced DNA damage in UV radiation-induced oxidative stress. In addition, caffeic acid showed *in vivo* antioxidant activity against chemical-induced toxicity (such as cisplatin-, carbon tetrachloride-, and cadmium-induced toxicity of the liver and kidney) in various animals (79).

Not only polyphenols but also other phytochemicals, vitamins and elements found in plants have also been reported to be responsible for antioxidant capacity. *T. indica* leaves contain the sugar acid form of ascorbic acid and some elements that possess antioxidant effects, e.g., selenium, copper, zinc, and manganese. These constituents might exhibit antioxidative effects via different mechanisms, and the overall antioxidant capacity might be caused by antagonistic, synergistic, or additional effects of these compounds and elements.

Most of the 21 studies that were reviewed executed antioxidant capacity by using more than one assay. This might be due to the differences in method principles and their strengths and limitations regarding cost and facility requirements, difficulty of operation, time spent, sensitivity and specificity, reproducibility and repeatability, correlation with phytochemical content, coverage spectrum of biological samples, and representativeness of the *in vivo* system. As a consequence of these factors, the results obtained from each study did not correlate with others.

Furthermore, the results still differed even when the same antioxidant assay was used. These occurrences were attributed to variations in the sources of the raw materials, their pretreatments, the extraction methods, and the solvents used for sample preparation. Furthermore, the phytochemical screening results differed between studies in that some classes of plant constituents were detected by some but not others. Other reasons for discrepancies in the screening results are that the quantity of the compounds was below the detection limit of the particular screening method employed and interference from other chemicals. Both factors might cause false-positive and false-negative results.

Considering toxicity, several studies performed the acute oral toxicity of *T. indica* leaf extracts, and no death was observed at the maximum single dose of 5,000 mg/kg BW and a 14-day repeated dose of 4,000 mg/kg BW. However, it was found that after exposure to *T. indica* leaf fluid extract in the right malar bag in Syrian hamsters, signs of mucous irritation were observed. These findings were explained by the presence of organic acids and polyphenols in *T. indica* leaves, which could slightly irritate the mucous membrane. Hence, the extract is considered a light irritant to the mucous membrane and could be a very mild irritant to the skin (49).

## Conclusions and future recommendations

In the present study, the antioxidant capacity of *T. indica* leaves was reviewed. *T. indica* leaf extracts exhibited *in vitro* antioxidant capacity through free radical scavenging capacity and transition and heavy metal chelating capacity. There is a high possibility that the antioxidant capacities are responsible for the polyphenols and the elements. The polyphenols found in *T. indica* leaves are flavonoids and phenolic acids such as catechin, vitexin, orientin, apigenin, and luteolin. In addition, elements such as selenium, copper, manganese, and zinc are present. These chemicals and elements are well-known as strong antioxidants, which makes *T. indica* leaves a promising natural antioxidant mixture. The safety of *T. indica* leaves was investigated in erythrocytes and animals. The extracts were found to be safe after oral administration of 4,000 mg/kg BW for 14 days, and no death was observed after the ingestion of 5,000 mg/kg BW. The 50% lethal intraperitoneal dose was 566 mg/kg BW.

The limitations of this systematic review are as follows:

- All studies were *in vitro-*based experiments.
- Positive controls were used only in some studies. Therefore, comparisons between the studies and the reported potency of *T. indica* leaf extracts are difficult to make.
- The extracts used in the included studies were not quantified for each active constituent or each biomarker of antioxidant capacity. The total phenolic and total flavonoid contents were shown in 17 studies.
- The maturity level of leaves and technology used in the treatment and extraction method were reported in only some studies.

To apply *T. indica* leaf extract as a source of antioxidant, confirmed results from an *in vivo* study and a clinical trial should be considered. Standardization of the extract with regard to its active constituents or total phenolic and total flavonoid content should be performed, especially if the extract is prepared using a different method and solvent. The effective dose should be taken into account to avoid excessive intake and antioxidative stress. The toxicity might also be a concern. An intensive *in vivo* study of subacute, subchronic, and chronic toxicity should be performed.

## Data availability statement

The original contributions presented in the study are included in the article/Supplementary material, further inquiries can be directed to the corresponding author/s.

## Author contributions

SS, AD, and SSa: conceptualization. SSo and SSa: data curation, formal analysis, and writing—original draft. SS, AD, and SSa: methodology. SSa, AD, and PP: supervision. AD and PP: validation. SSo, AD, SSa, and PP: writing—review and editing. All authors contributed to the article and approved the submitted version.

## Funding

This work was partially supported by the Unit of Excellence on Clinical Outcomes Research and IntegratioN (UNICORN) (grant number: FF65-UoE005), School of Pharmaceutical Sciences, University of Phayao and Phayao Provincial Public Health Office. The funding sources had no role in the study design or the collection, analysis, and interpretation of the data.

## Conflict of interest

The authors declare that the research was conducted in the absence of any commercial or financial relationships that could be construed as a potential conflict of interest.

## Publisher's note

All claims expressed in this article are solely those of the authors and do not necessarily represent those of their affiliated organizations, or those of the publisher, the editors and the reviewers. Any product that may be evaluated in this article, or claim that may be made by its manufacturer, is not guaranteed or endorsed by the publisher.

## Abbreviations

AAE: Ascorbic acid equivalent
ABTS, 2: 2'-azino-bis (3-ethylbenzthiazoline-6-sulfonic acid)
BCB: β-carotene bleaching
BHA: Butylated hydroxy anisole
BHT: Butylated hydroxytoluene
BW: Body weight
DPPH, 1: 1-diphenyl-2-picrylhydrazyl
DW: Dried weight
EDTA: Ethylene diamine tetraacetic acid
FE: Ferrous equivalent
FIC: Ferrous ion chelating
FRAP: Ferric ion reducing antioxidant power
GAE: Gallic acid equivalent
H_2_O_2_: Hydrogen peroxide
IC_50_: Half maximal inhibitory concentration
N/A: Data not available
NO: Nitric oxide
${\text{O}}_{2}^{•-}$: Superoxide radical
QE: Quercetin equivalent
ROS: Reactive oxygen species
RUE: Rutin equivalent
SD: Standard deviation
TE: Trolox equivalent
Temp: Temperature
T_room_: Room temperature.
