# Supplementary figures and images for: Botanical aspects, phytochemicals, and toxicity of Tamarindus indica leaf and a systematic review of antioxidant capacities of T. indica leaf extracts
Source: Front Nutr. 2022 Sep 20;9:977015. doi: 10.3389/fnut.2022.977015 (PMC9530316; doi:10.3389/fnut.2022.977015)

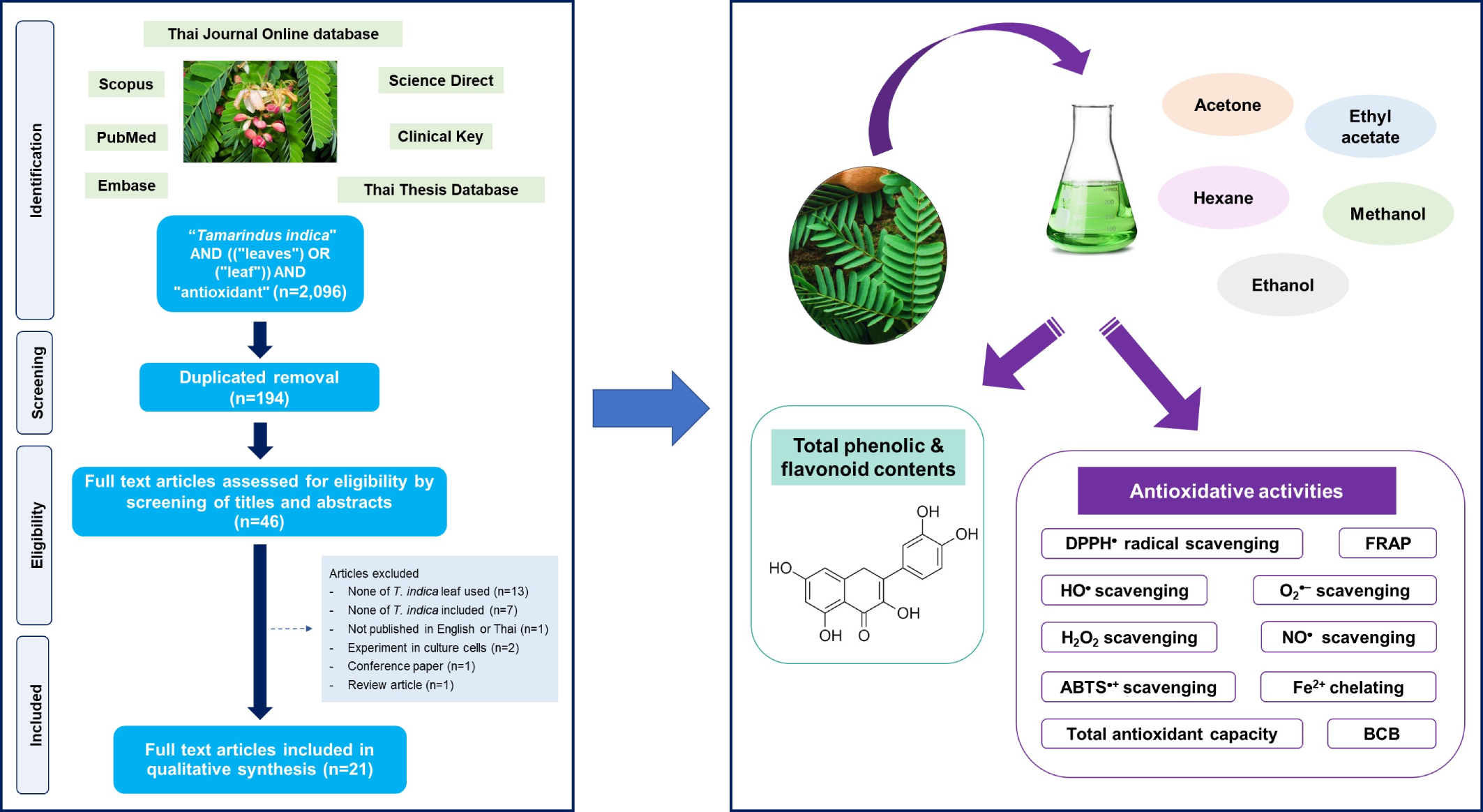

Supplement: Supplementary file 1 [file Image_1.tif]
